# Mechanism of splenic cell death and host mortality in a *Plasmodium yoelii* malaria model

**DOI:** 10.1038/s41598-017-10776-2

**Published:** 2017-09-05

**Authors:** Norinne Lacerda-Queiroz, Nicolas Riteau, Richard T. Eastman, Kevin W. Bock, Marlene S. Orandle, Ian N. Moore, Alan Sher, Carole A. Long, Dragana Jankovic, Xin-zhuan Su

**Affiliations:** 1 Laboratory of Malaria and Vector Research, National Institute of Allergy and Infectious Diseases, National Institutes of Health, Bethesda, Maryland 20892-8132 USA; 2 Laboratory of Parasitic Diseases, National Institute of Allergy and Infectious Diseases, National Institutes of Health, Bethesda, Maryland 20892-8132 USA; 3 Infectious Disease Pathogenesis Section of Comparative Medicine Branch, National Institute of Allergy and Infectious Diseases, National Institutes of Health, Bethesda, Maryland 20892-8132 USA

## Abstract

Malaria is a fatal disease that displays a spectrum of symptoms and severity, which are determined by complex host-parasite interactions. It has been difficult to study the effects of parasite strains on disease severity in human infections, but the mechanisms leading to specific disease phenotypes can be investigated using strains of rodent malaria parasites that cause different disease symptoms in inbred mice. Using a unique mouse malaria model, here we investigated the mechanisms of splenic cell death and their relationship to control of parasitemia and host mortality. C57BL/6 mice infected with *Plasmodium yoelii nigeriensis* N67C display high levels of pro-inflammatory cytokines and chemokines (IL-6, IFN-γ, TNF-α, CXCL1, and CCL2) and extensive splenic damage with dramatic reduction of splenic cell populations. These disease phenotypes were rescued in RAG2^−/−^, IFN-γ^−/−^, or T cell depleted mice, suggesting IFN-γ and T cell mediated disease mechanisms. Additionally, apoptosis was one of the major pathways involved in splenic cell death, which coincides with the peaks of pro-inflammatory cytokines. Our results demonstrate the critical roles of T cells and IFN-γ in mediating splenic cell apoptosis, parasitemia control, and host lethality and thus may provide important insights for preventing/reducing morbidity associated with severe malaria in humans.

## Introduction

Malaria, caused by *Plasmodium* parasites, is a deadly disease that infects hundreds of millions of people annually, leading to approximately 0.35 million deaths^1^. The major complications of the disease include cerebral malaria (CM), acute respiratory distress syndrome, acute renal failure, severe anemia, acidosis, hypoglycemia, hyper-reactive malarial splenomegaly syndrome (HMS), and toxic shock. Cerebral malaria has been linked to high plasma levels of inflammatory cytokines^2–4^ and/or expression of particular parasite antigens on the surface of infected red blood cells (iRBCs) and the sequestration of iRBCs in the brain^5^. Similarly, severe malaria anemia has been associated with clearance of uninfected red blood cells and/or suppression of hematopoiesis by high levels of inflammatory cytokines, particularly TNF-α and IFN-γ^6–10^.

The spleen is an important organ in clearing iRBCs, and malaria infection can lead to HMS and rupture of the spleen^11–16^. HMS is common in some tropical areas and is often associated with anemia^17^. *Plasmodium falciparum* malaria infection may also result in the impairment of splenic function known as a hyposplenic state that increases the risk of invasive bacterial infections^18^. Several studies have shown that infections of rodent malaria parasites, including *Plasmodium berghei* ANKA (PbANKA), *Plasmodium yoelii*, and *Plasmodium chabaudi* AS, could result in apoptosis of splenic T cells^19–21^. Further, IFN-γ was shown to be essential for apoptotic removal of T cell populations^22^. Nevertheless, the molecular mechanisms underlying malaria-induced splenic cell death and the subsequent effects of spleen malfunction on parasitemia control, inflammation, and host survival remain poorly understood and require additional investigations.

Studies have suggested that severe malaria is caused in large part by overreaction of host immune responses and/or metabolic disorders such as hypoglycemia and lactic acidosis, and that the risk of severe malaria is not directly related to risk of infection or frequency of exposure^23–28^. Individuals with low parasitemia can suffer from severe disease, whereas others having higher parasite loads remain asymptomatic^25, 29, 30^, suggesting that disease severity may not be always associated with blood parasite biomass. Activation of various subsets of T cells and secretion of pro-inflammatory cytokines, such as IFN-γ and TNF-α, have been shown to be critical for protection; however, over activation of T cells and cytokine production can also lead to severe disease^31, 32^. Production of IFN-γ by T cells has been associated with experimental cerebral malaria (ECM)^33–36^ and anemia^7^.

Using a model of *P. yoelii nigeriensis* N67C (N67C) infection in C57BL/6 mice, we previously showed that the host mounts a vigorous innate immune response, with massive loss of red pulp splenic cells by day 4 post-infection (*p.i*.)^37, 38^. The early death of infected mice at relatively low parasitemia (~40%, compared with mice infected with the YM strain that can survive >80% parasitemia) suggests that parasite burden is unlikely the direct cause of host death in this model. Here we systematically examined the effects of various immune cell populations in the spleen following N67C infection and the role of several immune mediators on parasite growth, splenic cell death, and host mortality. We demonstrate that IFN-γ production by T cells is associated with apoptosis of red pulp splenic macrophages (Mɸ), dendritic cells (DC) and natural killer (NK) cells, uncontrolled parasite growth, and eventually host death, providing a molecular and immunological basis for N67C-induced severe malaria.

## Results

### N67C induces a lethal inflammatory disease with massive splenic cell loss

**Figure 1:**
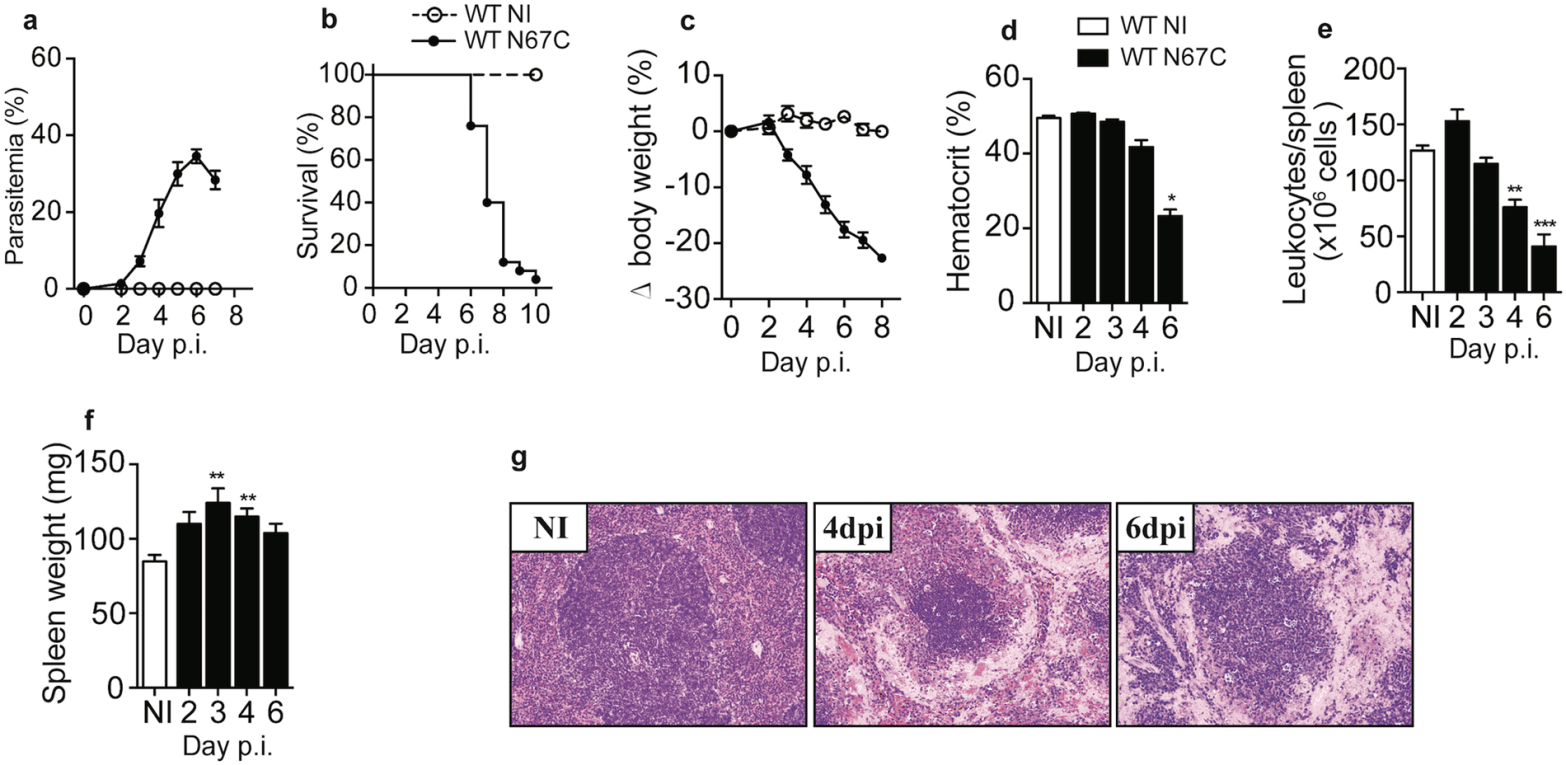
Kinetics of disease in C57BL/6 mice infected with the N67C parasite. (**a)** Parasitemia, **(b)** survival, **(c)** body weight, **(d)** hematocrit, **(e)** number of splenic leukocytes, and **(f)** total spleen weight over the course of N67C infection. **(g)** Representative photomicrographs of hematoxylin and eosin (H&E) stained spleen sections from non-infected (NI) and N67C-infected mice at days 4 and 6 post-infection (dpi) showing progressive loss of cellularity mainly in the red pulp and marginal zone (40X magnification). The results are expressed as mean ± SEM (3–5 mice) and are representative of two or three independent experiments. Kruskal-Wallis test, *p < 0.05, **p < 0.01, and ***p < 0.001 (compared to NI group).

Mice infected with N67C displayed a progressive increase in parasitemia in the early phase of infection, ultimately leading to host death by day 7–8 *p.i*. (Fig. 1a,b). Compared with uninfected mice, infected mice also had significant decrease in body weight (Fig. 1c), hematocrit (Fig. 1d), and splenic leukocytes (Fig. 1e) from day 4 *p.i*., although the total spleen weights were significantly increased at days 3 and 4 *p.i*. (Fig. 1f). Importantly, extensive loss of cellularity in the red pulp and marginal zone was also observed (Fig. 1g), which presents a unique feature for studying mechanism of potential splenic cell death.

In addition, histopathological analyses of lung tissues from infected mice also revealed pulmonary alterations associated with acute respiratory distress day 6 *p.i*., including thickening of the alveolar wall, mononuclear cell infiltration, and edema (Supplementary Fig. S1a). Increases in leukocyte number and protein level in the bronchoalveolar lavage (BAL) fluid of infected mice were also observed (Supplementary Fig. S1b,c). Moreover, significantly higher levels of Evans Blue were detected in the spleen and lung parenchyma, but not in the brain, of infected mice day 6 *p.i*. (Supplementary Fig. S1d), indicating vascular leakage and endothelial dysfunction. These data suggest a systemic inflammatory process causing dramatic damage in the lungs and possibly other organs in addition to the spleen following N67C infection.

### N67C infection triggers early reductions in splenic cell populations

**Figure 2:**
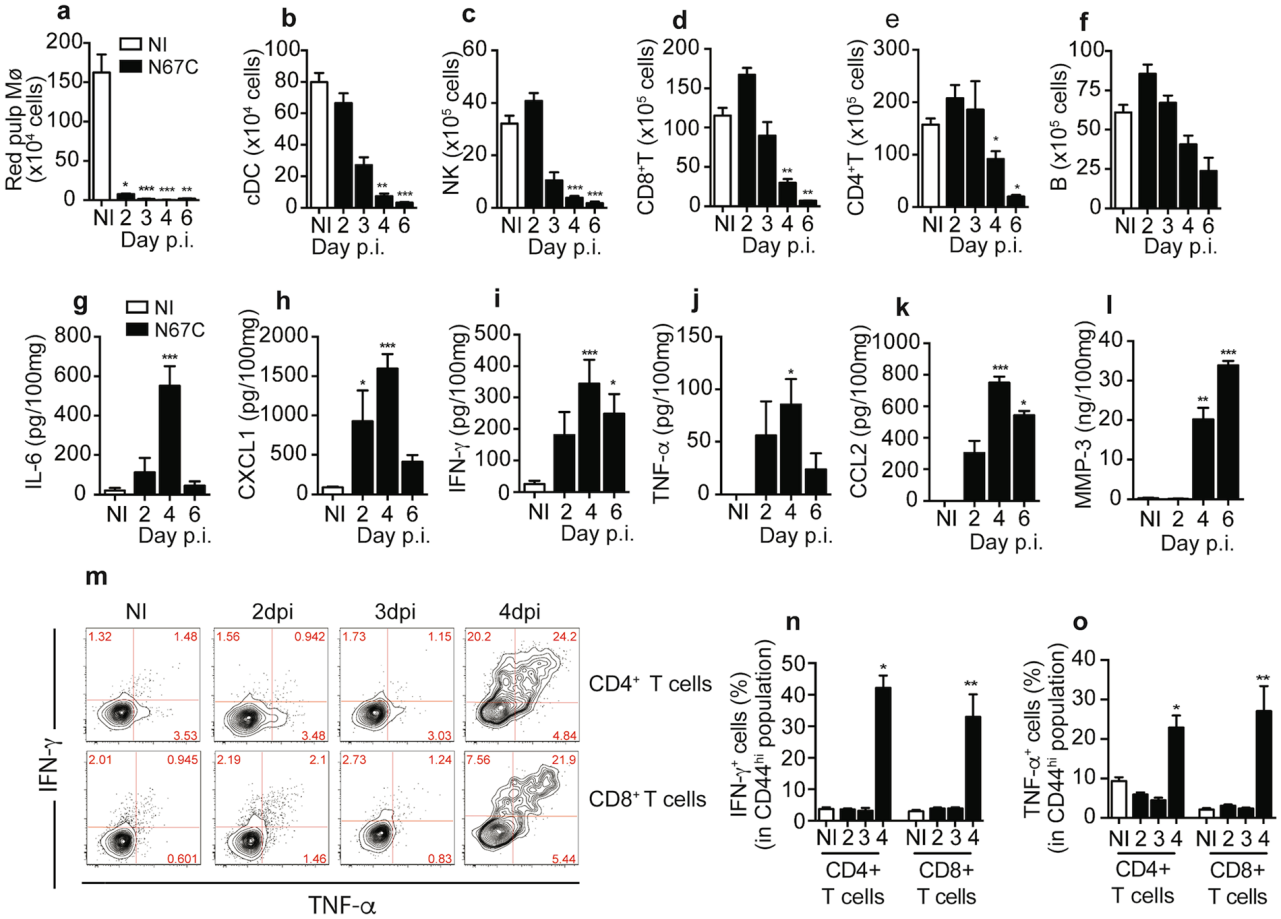
Kinetic of immune cell populations and levels of cytokines and chemokines in the spleen of N67C-infected mice. Cell counts of red pulp macrophages (Mɸ) from uninfected (NI) and N67C-infected spleens days 2–6 *p.i*. **(a)**, of conventional dendritic cells (cDC) **(b)**, natural killer (NK) cells **(c)**, CD8 + T cells **(d)**, and CD4 + T cells **(e), B lymphocytes (f)**. Cells were counted using flow cytometry after staining with cell-specific markers. **(g–l)** Pro-inflammatory mediators in homogenates of spleens, measured using ELISA. **(m)** Representative flow cytometry plots of CD4^+^ CD44^hi^ and CD8^+^ CD44^hi^ splenic T cells of uninfected (NI) and N67C-infected mice expressing intracellular TNF-α and IFN-γ. Splenocytes were stimulated *ex vivo* with anti-CD3/CD28 in the presence of Brefeldin A before staining. dpi, day post infection. **(n,o)** Percentage of TNF-α or IFN-γ positive CD4+ and CD8+ splenic T cells day 2, 3 and 4 *p.i*., after infection with N67C parasite. The results are expressed as mean ± SEM (3–5 mice) and are representative of two or three independent experiments. Kruskal-Wallis test, *p < 0.05, **p < 0.01, and ***p < 0.001 (compared to NI group).

Flow cytometric analyses were performed to characterize changes in spleen cell populations after N67C infection. Compared with those of uninfected mice, significant reductions in the number of red pulp Mɸ from day 2 *p.i*. (Fig. 2a) and conventional DC (cDC), NK cells, CD8+ and CD4+ T cells from day 4 *p.i*. were observed over the course of infection (Fig. 2b-e; Supplementary Fig. S2). The number of B cells was also reduced, but not significant (Fig. 2f). Accordingly, we observed significant reductions in the percentages (%) of red pulp Mɸ from day 2 *p.i*., NK cells from day 3 *p.i*., cDC from day 4 p.i. and CD8+ T cells on day 6 p.i., but no reduction in the percentages (%) of B and CD4^+^ T cells was obtained (Supplementary Fig. S3a–f). These results suggest splenic cell death, although we cannot rule out the possibility that migration of immune cells from the spleen to other tissues contributes to the decrease in spleen cellularity. The reduction in cell count is consistent with the histopathological observations of the loss of cellularity in the red pulp of the spleen on days 4 and 6 *p.i*.

### Levels of pro-inflammatory cytokines are increased after N67C infection

We next measured pro-inflammatory mediators in splenic tissue by ELISA and observed significant increases in IL-6, CXCL1(KC), IFN-γ, TNF-α, CCL2 (MCP-1), and MMP-3 levels after infection (Fig. 2g–l). Kinetic analyses of IFN-γ and TNF-α production by activated CD44^hi^ spleen T cells following *in vitro* stimulation with anti-CD3/CD28 in the presence of Brefeldin A showed high frequencies of IFN-γ and/or TNF-α containing CD4+ and CD8+ T cells at day 4 *p.i*. (Fig. 2m–o). These results suggest that splenic CD4+ and CD8+ T cells could be the major sources of IFN-γ and TNF-α, and that the early death of NK, Mɸ, and T cells might play an important role in immune-mediated pathology during N67C infection.

### Infected RAG2^−/−^ mice display improved host survival and reduced splenic cell death

**Figure 3:**
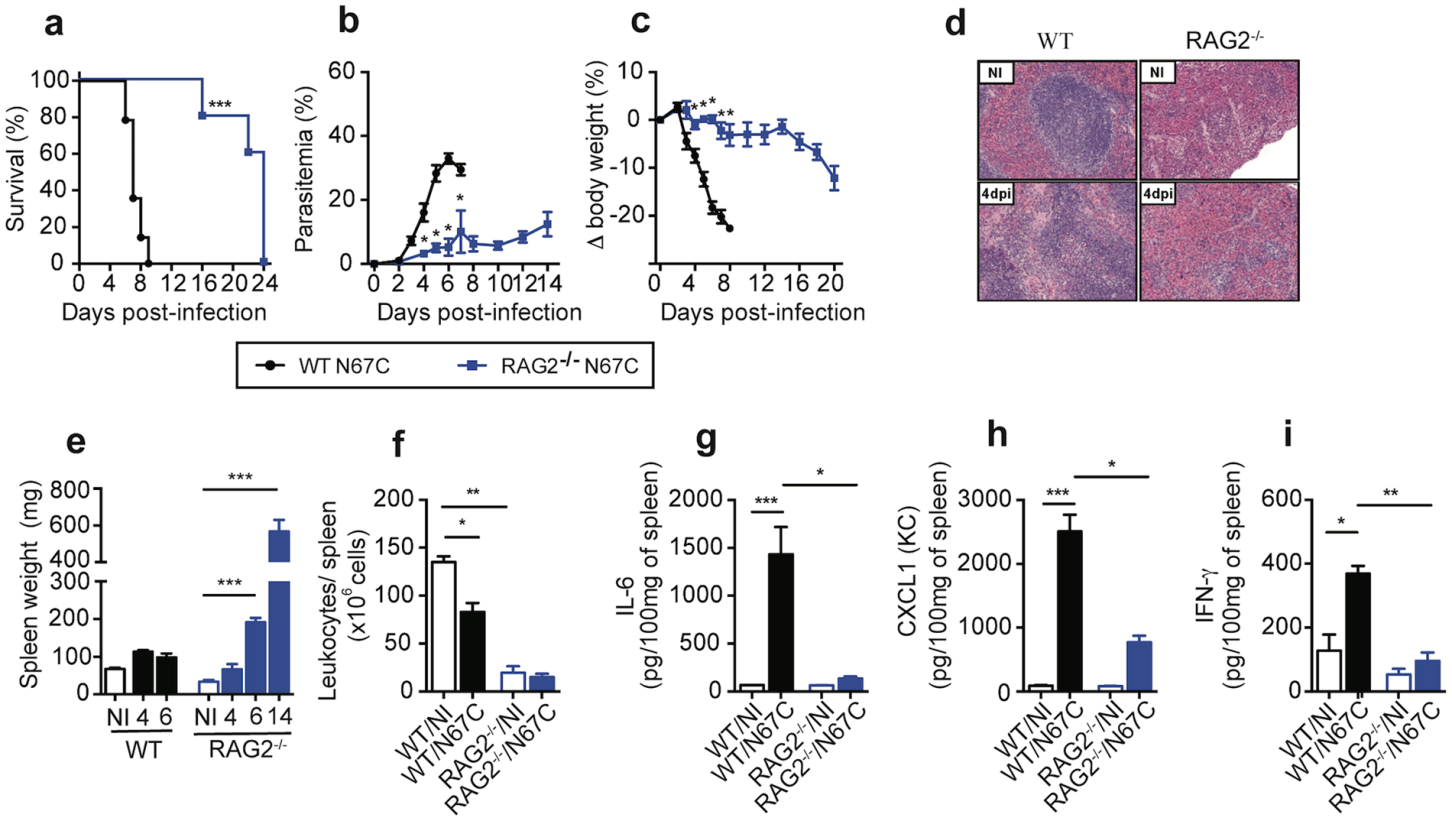
N67C-infected RAG2 deficient mice display less severe disease phenotypes and reduced cytokine production. **(a)** Host survival, **(b)** parasitemia, and **(c)** body weight variation of wild type (WT) and RAG2^−/−^ mice after N67C infection. **(d)** Representative photomicrographs of hematoxylin and eosin (H&E) stained spleen tissues (40X magnification) from non-infected (NI) and infected WT or RAG2^−/−^ mice at day 4 *p.i*. **(e,f)** Spleen weight **(e)** and numbers of splenic leukocytes (**f)** of uninfected (NI) or N67C infected spleens from WT or RAG2^−/−^ mice. **(g–i)** Levels of IL-6, CXCL1(KC), and IFN-γ from the spleen tissues of uninfected or infected WT and RAG2^−/−^ mice day 4 *p.i*. Data are mean ± SEM (3**–**5 mice) and are representative of two or three independent experiments; Kruskal-Wallis test, *p < 0.05, **p < 0.01, and ***p < 0.001.

Since CD4^+^ and CD8^+^ T cells were found to be the major producers of IFN-γ and TNF-α, we infected RAG2^−/−^ mice that did not have functional B and T cells to investigate the roles of lymphocytes in the N67C-induced splenic cellular loss and host mortality. Infected RAG2^−/−^ mice had significantly lower parasitemia, better survival rate, and lesser body weight loss compared with those of infected WT mice (Fig. 3a–c). Eventually, the infected RAG2^−/−^ mice succumbed to infection after day 20 *p.i*. Even though naïve RAG2^−/−^ mice have substantial differences in their spleen architecture, size, and cellularity due to the absence of lymphoid cells, spleen sections of the RAG2^−/–^ infected mice did not show loss of cellularity, in sharp contrast with those of WT-infected mice (Fig. 3d). In addition, infected RAG2^−/−^ mice had significantly larger spleens on day 6 and 14 *p.i*., but not on day 4 *p.i*., than those of non-infected mice (Fig. 3e). This prominent splenomegaly was associated with extramedullary hematopoiesis (EMH) as indicated by histological analysis, although we cannot rule out the possibility of myeloid progenitors released from bone marrow. Furthermore, on day 4 *p.i*., the total numbers of splenic leukocytes in infected RAG2^−/−^ mice were similar to those of the naïve RAG2^−/−^ group (Fig. 3f). Spleen cells from infected RAG2^−/−^ mice also produced significantly lower levels of IL-6, CXCL1, and IFN-γ as compared to infected WT mice (Fig. 3g–i). These results suggest that lymphocytes and/or adaptive immunity play an important role in disease severity, spleen pathology, and production of inflammatory cytokines/chemokines during malaria infection. The preservation of red pulp phagocytic cells may contribute to better control of parasitemia in the RAG2^−/−^ mice than in WT mice. This lower parasitemia in N67C-infected RAG2^−/−^ mice is in contrast to higher parasitemia in RAG KO mice infected with *P. chabaudi* ^39^, suggesting different mechanisms of parasitemia control in mice infected with these two parasite species.

### T cell depletion leads to improved host survival and reduced splenic cellular loss

**Figure 4:**
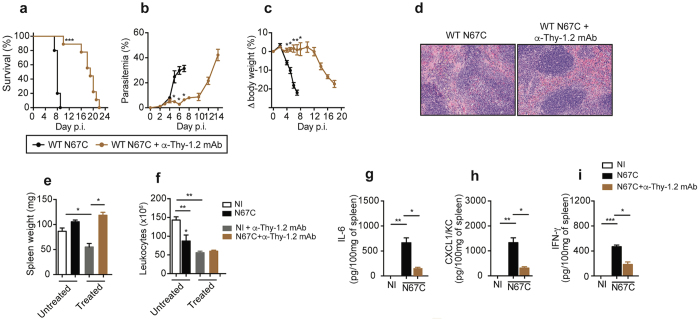
Effects of T cell depletion on parasitemia, host survival, spleen pathology, and cytokine responses. **(a)** Host survival, **(b)** parasitemia, **(c)** body weight changes of WT and WT mice treated with anti-Thy-1.2 mAb after infection with N67C. **(d)** Representative hematoxylin and eosin (H&E) stained spleen tissues (40X magnification) from WT or anti-Thy1.2 treated mice after N67C infection. **(e** and **f)** Spleen weight **(e)** and total leukocyte number **(f)** in uninfected (NI) or infected mice with or without mAb treatment. **(g–i)** Levels of IL-6 **(g)**, CXCL1 **(h)**, and IFN-γ **(i)** in the spleens of uninfected or infected mice. Data are mean ± SEM (3**–**5 mice) and are representative of two independent experiments; Kruskal-Wallis test, *p < 0.05, **p < 0.01, and ***p < 0.001.

To further investigate the potential role of T cells in the disease phenotypes, we depleted T cells in WT mice using anti-Thy-1.2 mAb (CD90.2) before and during the course of N67C infection. The anti-Thy-1.2-treated mice had higher survival rates, lower parasitemia, and less body weight loss compared to untreated WT mice (Fig. 4a–c). In contrast to WT-infected mice, anti-Thy-1.2-treated mice showed no obvious signs of splenic cellular loss (Fig. 4d), suggesting amelioration of disease. The treatment for T cell depletion in naïve WT mice *per se* resulted in reduction of leukocyte number; however, the infected mice had larger spleens than uninfected mice, without any reduction in the total number of splenic leukocytes between the two groups (Fig. 4e,f). Interestingly, the levels of IL-6, CXCL1, and IFN-γ were significantly reduced in the anti-Thy-1.2-treated mice than in those of untreated mice (Fig. 4g–i).

Additional cell depletion experiments were performed to assess the relative contributions of other immune cell types during N67C infection. However, depletion of NK cells (anti-NK1.1, clone PK136), neutrophils (anti-Ly6G mAb, clone 1A8), or neutrophils and Ly6C^+^ monocytes (anti-Gr1 mAb, clone RB6–8C5) did not significantly alter survival rates or spleen pathology as compared to untreated mice (data not shown). Altogether, these results strongly support an important role of T cells in splenic cell loss and host disease phenotypes.

### IFN-γ depletion significantly reduces disease severity and cytokine levels

**Figure 5:**
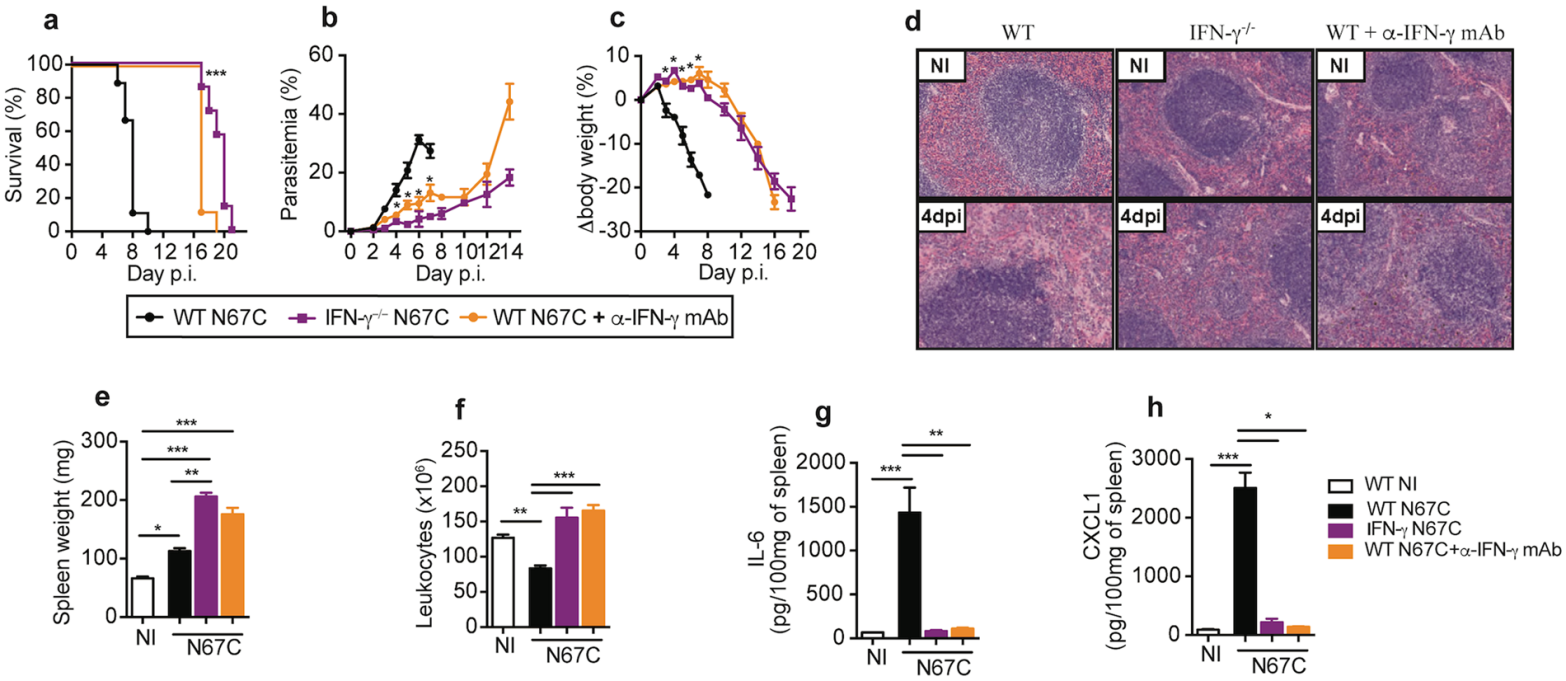
Absence of IFN-γ signaling recapitulates disease phenotypes as observed in RAG2^−/−^ mice. Wild type (WT), IFN-γ^−/−^, or IFN-γ depleted mice after treatment with anti-IFN-γ mAb (500 μg/mouse each on day −1, 1, 3, 5, and 7) were infected with N67C parasite, and host survival **(a)**, parasitemia **(b)**, and body weight change **(c)** were measured after infection. **(d)** Representative photomicrographs of hematoxylin and eosin (H&E) stained spleen sections (40X magnification) from non-infected (NI) and N67C-infected WT, IFN-γ^−/−^, and anti-IFN-γ mAb treated mice at day 4 *p.i*. **(e,f)** Spleen weight and total splenocyte numbers of WT, IFN-γ^−/−^, or anti-IFN-γ mAb treated mice at day 4 *p.i*. **(g,h)**. Levels of IL-6 and CXCL1 in spleen tissues from non-infected (NI) and N67C-infected WT, IFN-γ^−/−^, and anti-IFN-γ mAb treated mice at day 4 *p.i*. measured using ELISA. The results are expressed as mean ± SEM (3**–**5 mice) and are representative of two experiments; Kruskal-Wallis test, *p < 0.05, **p < 0.01, and ***p < 0.001.

The ability of T cells to drive immunopathology during N67C infection led us to investigate the potential role of IFN-γ, a well-described Th1 cytokine produced by T and other cells with contradictory roles in *Plasmodium* infections^32^. We first evaluated disease outcomes in IFN-γ^−/−^ and anti-IFN-γ mAb-treated WT mice. Both IFN-γ-deficient and IFN-γ-depleted mice survived longer than WT control mice, displaying significantly lower parasitemia and higher body weight than those of WT mice (Fig. 5a–c). On day 4 *p.i*., the absence of IFN-γ signaling resulted in minimal signs of cellular loss in the spleen tissue (Fig. 5d), as well as enlarged spleen with no reduction in splenocyte numbers (Fig. 5e,f). The levels of IL-6 and CXCL1 in the spleen tissue were also significantly reduced (Fig. 5g,h). Splenomegaly and EMH were predominant in IFN-γ^−/−^ mice, as observed at day 6 and 14 *p.i*. (Supplementary Fig. S4a,b).

**Figure 6:**
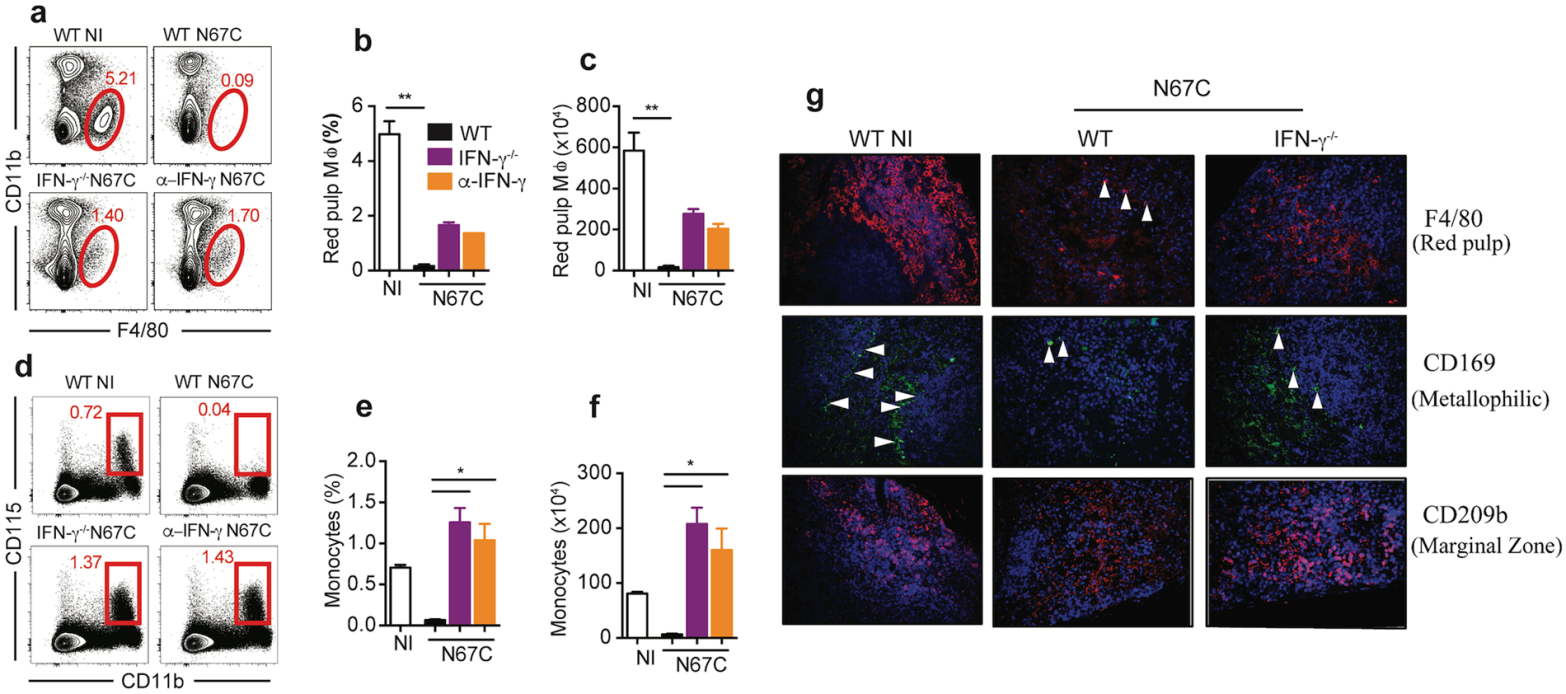
Retention of macrophages (Mɸ) subtypes and monocytes in IFN-γ^−/−^ mice. **(a)** Representative flow cytometry plots of red pulp Mɸ (F4/80^+^ CD11b^-^) of WT, IFN-γ^−/−^ and anti-IFN-γ mAb treated mice day 4 *p.i*. **(b,c)** Percentage and absolute number of red pulp Mɸ. **(d)** Representative flow cytometry plots of inflammatory monocytes (CD11b^+^ CD115^+^). (**e,f**) Percentage and absolute number of inflammatory monocytes. Data in **(b,c** and **e,f)** are mean ± SEM (3**–**5 mice) and are representative of two experiments; Kruskal-Wallis test, *p < 0.05, and ***p < 0.01. **(g)** Immunofluorescent staining of splenic Mɸ sub-populations using specific markers as described in^40^. The red pulp Mɸ was stained by F4/80 expression, marginal zone Mɸ by CD209b, and metallophilic Mɸ by CD169 (40X magnification). The arrowheads point to stained cells of different sub-populations of macrophages.

In addition, IFN-γ depletion partially prevented the loss of red pulp Mɸ in infected WT mice at day 4 *p.i*. (Fig. 6a–c) and greatly increased the frequency and absolute number of inflammatory monocytes in the spleen tissue during N67C infection (Fig. 6d–f). Different splenic Mɸ populations are present at distinct anatomical locations and can be identified using specific surface markers^40^. By immunofluorescence, red pulp Mɸ can be identified by F4/80 expression, metallophilic Mɸ by CD169, and marginal zone Mɸ by CD209b (or SIGN-R1). Immunofluorescence staining of infected spleen tissues revealed the maintenance of marginal zone and metallophilic Mɸ in IFN-γ^-/–^infected mice at day 4 *p.i*. (Fig. 6g). In addition, the absence of IFN-γ largely prevented the loss of CD8 + T cells, NK cells, and cDCs (Supplementary Fig. S4c–g) and led to a decreased TNF-α production by activated CD44^hi^ CD4+ or CD8+ T cells (Supplementary Fig. S4h–j **)**. Collectively, these findings demonstrate that the absence of IFN-γ reduces the loss of splenic macrophages and enhances host survival during N67C infection.

### N67C infection leads to increased signals of apoptosis

**Figure 7:**
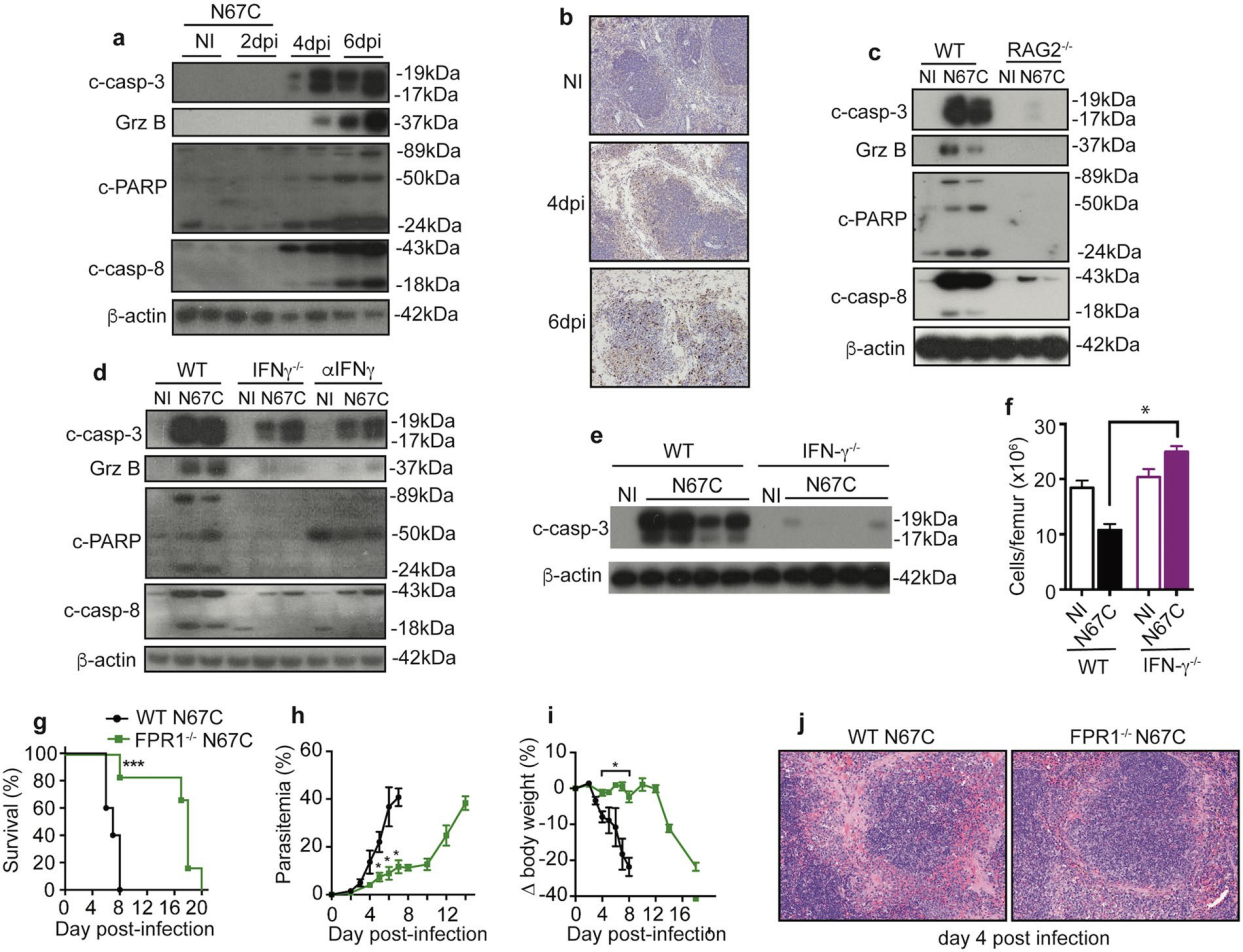
Lymphocyte and IFN-γ mediated apoptosis as one of the major pathways of splenic cell death. Total spleen tissue homogenates were analyzed by Western blot using antibodies as indicated. **(a)** Detection of cleaved caspase-3, cleaved caspase-8, cleaved PARP, and granzyme B (GrzB) in spleen tissue homogenates of WT naïve (NI) and N67C infected mice at days 2, 4, and 6 *p.i*. **(b)** Immunostaining of spleen sections for cleaved caspase-3. **(c)** Detection of cleaved caspase-3, cleaved caspase-8, cleaved PARP, and granzyme B at day 4 *p.i*. in spleen tissues of WT and RAG2^−/−^ mice. **(d)** Detection of cleaved caspase-3, cleaved caspase-8, cleaved PARP, and granzyme B in the spleen tissues of WT, IFN-γ^−/−^ or mice treated with anti-IFN-γ mAb. **(e)** Signals of cleaved caspase-3 in bone marrow (BM) of naïve (NI) or infected WT and IFN-γ^−/−^ mice. **(f)** Total cell numbers from BM femurs of WT and IFN-γ^−/−^ mice. Data are mean ± SEM (3**–**5 mice) and are representative of two independent experiments. (**g–j**) Host survival (**g**), parasitemia (**h**), body weight (**i**), and representative photomicrographs of hematoxylin and eosin (H&E) stained spleen sections (**j**) from N67C-infected WT and FPR1^−/−^ mice (40X magnification). Kruskal-Wallis test, *p < 0.05, ***p < 0.001. Gels for **a**, **c**, **d**, and **e**, were run under the same experimental conditions, and the figures were cropped from original gel images. The original Western blot images can be found in Supplementary Fig. 5.

To investigate potential pathways of cellular death, we first compared apoptotic markers in the spleen lysates of WT mice. In contrast to naïve animals, N67C infection leads to increased levels of cleaved caspase-3, cleaved caspase-8, cleaved PARP, and granzyme B (GrzB) on days 4 and 6 *p.i*. (Fig. 7a and Supplementary Fig. S5 **)**. Immunostaining of spleen sections for cleaved caspase-3 also showed increased numbers of positive cells in the red and white pulp on days 4 and 6 *p.i*. (Fig. 7b **)**. The signals for cleaved caspase-3, cleaved caspase-8, cleaved PARP, and granzyme B were absent or greatly reduced in the spleen tissue of infected RAG2^−/−^ (Fig. 7c and Supplementary Fig. S5) and IFN-γ^−/−^ mice (Fig. 7d and Supplementary Fig. S5), suggesting that lymphoid cells and IFN-γ play a direct role in splenic cell apoptosis. Similarly, a reduction in cleaved caspase-3 signal was observed in bone marrow (BM) cells of N67C infected IFN-γ^−/−^ mice (Fig. 7e and Supplementary Fig. S5). The number of total BM cells was also increased in the IFN-γ^−/−^ mice compared with those of infected WT mice (Fig. 7f). These results demonstrate that apoptosis is one of the pathways mediating splenic cell loss after N67C infection.

We also considered the possible role of other cell death pathways including caspase-1 dependent pyroptosis and various caspase independent pathways, such as necroptosis and ferroptosis^41^. We investigated the potential role of necroptosis and ferroptosis in disease severity by treating N67C infected WT mice with necrostatin-1 (Nec-1, an inhibitor of RIP1^42^), GW806742X (a p-MLKL inhibitor), and ferrostatin-1 (an inhibitor of lipid peroxidation in the ferroptosis pathway). No significant differences in host mortality or body weight among treated or untreated mice were observed (Supplementary Fig. S6a,b). We next infected caspase-1/caspase-11 double deficient mice with N67C, and again did not detect any differences in host mortality, parasitemia, or body weight between KO and WT mice (Supplementary Fig. S6c–e), suggesting that inflammasome-mediated cell death or pyroptosis might not play a significant role in disease severity and pathology in N67C infection. Recently, interaction of annexin A1 and formyl peptide receptor 1 (FPR1) was shown to contribute to necroptosis of keratinocytes in Stevens-Johnson syndrome (SJS) and toxic epidermal necrolysis (TEN)^43^. We also infected WT mice and mice lacking the *fpr1* gene and showed significantly higher survival rate, lower parasitemia, less body weight loss, and reduced splenic cell death in the FPR1-deficient mice than WT mice (Fig. 7g-j), suggesting that this specific necroptosis pathway mediated through interaction between annexin A1 and FPR1 may play a role in the N67C-induced cell death.

## Discussion

One of the unique features associated with N67C infection is massive splenic cell loss from day 4 *p.i*. Malaria-induced HMS and rupture of the spleen in human infections^11–14^ and T cell apoptosis after infections with various rodent malaria parasites have been reported previously^19–22^. Additionally, cellular losses of marginal and metallophilic macrophages were observed during *P. chabaudi* infection, and CD8 + T cell was implicated in the *P. chabaudi*-induced loss of the metallophilic macrophages^44^. Activation of the innate immune system, local expansion of monocytic cells, and the removal of iRBC have been attributed to the disruption of the splenic microarchitecture and splenomegaly in human and rodent malaria infections^45–47^, and the resolution of acute parasitemia during *P. chabaudi* infection has been associated with release of early myeloid progenitors from the BM and establishment of extramedullary splenic myelopoiesis^48, 49^. In addition to phagocytosis of the iRBCs, the spleen can also remove intraerythrocytic malaria parasites without destruction of the RBCs in acute human malaria infections^15, 50^. In a systemic pathological study of cerebral malaria in African children, enlarged spleens and abundant malaria pigments in splenic macrophages were observed in most of the 103 fatal patients^51^. These observations point to an important role of the spleen in parasite control and disease resolution. Dissecting the molecular mechanisms of splenic cell death observed in N67C infection may provide critical information for studying pathogenesis of human malaria and for prevention of host death. Using the N67C model that produces inflammatory lethal infection, here we investigate the molecular and immunological mechanisms underlying host responses and disease severity, particularly cellular loss in spleen red pulp. We determined which splenic cell populations were significantly affected by N67C infection and showed that red pulp Mɸ were almost completely depleted day 2 *p.i*., followed by significant reduction of cDC and NK cells on day 4 *p.i*. The early death of Mɸ, cDC, and NK cells suggest that some mechanisms of innate immunity such as phagocytosis of iRBCs were compromised. In contrast, the numbers of B and T cells were increased day 2 *p.i*., despite not significantly, before significant reduction on day 4 or day 6 *p.i*. These results show early activation and expansion of lymphocytes that may play a critical key role in the splenic cell death and disease severity in N67C infection. The signals of apoptosis and necroptosis in the spleen and bone marrow tissues, which may include NK, DC and Mɸ, after N67C infection add new information to the previously reported T cell apoptosis and CD8 + T cell mediated loss of the metallophilic macrophages in *P. chabaudi* infection^19–22, 44^.

We investigated the roles of T cells in splenic cell death, parasite growth, and host survival after N67C infection. T cells and NK cells are known to be the major sources of IFN-γ in many infections^32, 52–54^. Our data indicate that NK cells are not essential for the pathogenicity mediated by IFN-γ during early N67C infection because depletion of NK cells did not alter host mortality. T cells can be protective or pathogenic in malaria infections. For example, IFN-γ producing CD4^+^ T cells and CD4^+^ T cell help to promote B lymphocyte responses are required for the control and elimination of iRBCs^31^, and the numbers of circulating IFN-γ producing CD4 + T cells were positively correlated with protection in a human volunteer study^55^. In our N67C model, we showed that T cells played a key role in IFN-γ production, splenic cell apoptosis and FPR1-mediated necroptosis, and host death. Activated T cells likely become the major sources of IFN-γ and TNF-α on day 4 *p.i*. Indeed, the detrimental roles of T cells in host death and spleen immunopathology were clearly demonstrated in the experiments with RAG2^−/−^ mice or T cell depleted WT mice. N67C-infected RAG2^−/−^ mice had significantly lower parasitemia, higher body weight, and longer survival time than the infected WT mice. Importantly, splenic cell loss was completely reversed in the RAG2^−/−^ mice, and the levels of IL-6, IFN-γ, and CXCL1, were also significantly lower in the RAG2^−/−^ mice than in WT mice. Treatment of mice with anti-Thy1.2 mAb to deplete T cells also led to phenotypic measurements similar to those of RAG2^−/−^ mice.

Our study identifies IFN-γ as a key cytokine mediating cell death of splenic red pulp and host lethality. As mentioned before, IFN-γ can be protective or pathogenic in malaria infections. IFN-γ produced by activated T cells has been shown to be critical for immune protection in various studies^22, 32, 56–61^. Mice deficient in IFN-γ signaling have a delayed parasitemia resolution^62, 63^, and IFN-γ and IFN-γ-induced chemokines are required for mobilization of myeloid progenitors to the spleen during *P. chabaudi* infection^49^. Elevated levels of IFN-γ are also correlated with natural resistance to *P. falciparum* infection in the Malian Fulani population^56^. Similar to T cell function, excessive up-regulation of pro-inflammatory cytokines can also contribute to pathology and severe malaria^33–36, 64^. Disruption of the blood-brain barrier by CD8 + T cell-derived perforin and granzyme is likely the cause of experimental cerebral malaria (ECM) during PbA infection^33, 34^, and IFN-γ is necessary for the recruitment of CD8 + T cells to the brain during ECM^35^. In another study, IFN-γ induced endothelial ICAM-1 up-regulation and subsequent microvascular pathology after PbA infection, resulting in fatal ECM^65^. In our N67C model, we demonstrate that IFN-γ produced by T cells is a key cytokine causing splenic cell death (mostly NK, DC and Mɸ) and host mortality following N67C infection. IFN-γ^−/−^ mice and mice treated with anti-IFN-γ had disease phenotypes similar to those of RAG2^−/−^ mice and mice depleted of T cells, demonstrating that IFN-γ is a major trigger of splenic cell death and host lethality. Significantly higher levels of pro-inflammatory mediators such as IL-6, CXCL1, IFN-γ, TNF-α, CCL2, and MMP-3 were observed at day 4 *p.i*. in WT infected mice. We evaluated the effects of T cell and IFN-γ on production of cytokine and chemokines. RAG2^−/−^ mice, T cell depletion, and deficiency of IFN-γ also significantly reduced the levels of IL-6 and CXCL1, although we did not investigate the effects of these cytokine depletions on splenic cell death and host fatality directly. Additionally, infected mice with deficiency of RAG2, T cells, or IFN-γ had splenomegaly associated with EMH, which may represent a hematopoietic pathway in response to the loss of RBCs in these mice.

Since IFN-γ may act directly on Mɸ that play a key role in capturing iRBCs from the circulation, we investigated the profile of Mɸ after N67C infection. Splenic Mɸ present distinct anatomical locations and display specific markers^40^. Based on specific marker expression, we observed a significant loss of different Mɸ subtypes in WT-infected mice, but the maintenance of marginal zone and metallophilic Mɸ in IFN-γ^-/–^infected mice at day 4 *p.i*. Our results were consistent with the reports of the loss of marginal zone cells in *P. chabaudi* infection^44, 66, 67^. These results suggest that T cells exert their effects mostly through secretion of IFN-γ, and that IFN-γ signaling contributes to disease phenotypes.

Finally, we investigated the signaling pathways of splenic cell death after N67C infection. Strong signals of apoptotic markers, including cleaved caspase-3, cleaved caspase-8, cleaved PARP, and granzyme B were detected in spleen and BM tissues on day 4 and/or day 6 *p.i*. These signals of apoptosis disappeared or were greatly reduced in the RAG2^−/−^ mice and mice without IFN-γ, suggesting that IFN-γ, possibly together with TNF-α, play a direct role in splenic cell apoptosis. Induction of apoptosis by TNF-α is well-known^68^, and IFN-γ can also mediate or enhance apoptosis in other diseases or disorders^69^. However, we cannot rule out the involvement of other mechanisms of cellular death such as necroptosis and pyroptosis in splenic cell death^70^. We detected a weak 50 kDa band of cleaved PARP that is considered a maker for necrosis^71^, and FPR1^−/−^ mice had greatly reduced splenic cell death, although treatment of mice with necrostatin-1/GW806742X (p-MLKL inhibitor)/ferrostatin-1 and infection of caspase1/caspase11 double KO mice did not significantly change host survival rate. Our results suggest that both caspase mediated apoptosis and FRP-mediated necroptosis pathways contribute to cell death in the N67C infected mice. Additional investigations are necessary to clarify the molecular basis of N67C-induced cell death.

This study presents a comprehensive investigation of immune elements that contribute to malaria-induced spleen apoptosis and host lethality. Based on our observations, we can assemble some key molecular events leading to immune cell apoptosis and host death after N67C infection of C57BL/6 (Supplementary Fig. S7): N67C infection results in an early death of red pulp Mɸ, NK and cDC cells in the spleen and activation of T cells that produce IFN-γ and TNF-α leading to the extensive cellular death in the spleen, lungs, BM, and possibly other tissues. We detected strong signals for apoptotic markers in the spleen and in the BM; however, we cannot totally rule out the involvement of necroptosis and other cell death pathways. During the erythrocytic stage infection, the spleen is a key site for controlling malaria parasitemia, responsible for removal of iRBCs and development of protective and/or pathological immune responses. Mɸ and DC cells are the major phagocytic cells that engulf iRBCs, leading to the decline of parasitemia. The destruction of these cells early in N67C infection impairs the ability to control early parasite growth. Because N67C-infected mice died at ~40%–50% parasitemia, RBC destruction by rupture of iRBCs is unlikely to be the direct cause of host death. Although splenic cell apoptosis and high levels of IFN-γ, IL-12, TNF-α, and other cytokines might contribute to host lethality, the direct causes of severe disease and host death are likely due to multiple factors, including excessive inflammatory responses, a compromised immune system, organ failure, and metabolic disturbances such as hypoglycemia and lactic acidosis that we did not study here^24^. Our study investigates potential mechanisms of splenic cell death and host mortality mediated by T cells and IFN-γ in a mouse malaria model. The effects of T cells and IFN-γ on the splenic cell death and disease severity during *Plasmodium* infection in humans could be different from what we have observed in this mouse model and require additional investigations. Human infections generally have low parasitemia and are likely chronic than acute, although splenomegaly or rupture of spleen in human malaria patients are frequently reported^72–74^. Intervention in the overproduction of IFN-γ and pro-inflammatory mediators early in malaria infection may provide an important approach to treatment of severe malaria.

## Methods

### Ethics statement

All animal procedures for this study were performed in accordance with the protocol approved (approval #LMVR11E) by the Institutional Animal Care and Use Committee (IACUC) at the National Institute of Allergy and Infectious Diseases (NIAID) following the guidelines of the Public Health Service Policy on Humane Care and Use of Laboratory Animals and AAALAC. All mice were maintained under pathogen-free conditions.

### Mice with various gene deficiencies

Inbred female C57BL/6 and gene knockout (KO) mice of matched genetic background, aged 6–10 weeks old, were purchased from Charles River Laboratories International, Inc. (Frederick, Maryland, USA) or Jackson Laboratory (Bar Harbor, Maine, USA), or were obtained from the NIAID/Taconic repository. To block a specific ligand-receptor interaction, monoclonal antibodies (mAbs) were administered intraperitoneally (*i.p*.) into C57BL/6 mice every two days, starting one day before infection until day 7 post-infection (*p.i*.). To neutralize IFN-γ, mAb (XMG-1.2, Bio X Cell, West Lebanon, USA) was injected into C57BL/6 mice (500 µg/mouse). Depletion of lymphoid cells was performed using mAb against Thy-1.2 (30H12, Bio X Cell) (250 µg/mouse). Depletion efficiency of cell subtypes was confirmed by flow cytometry.

### Malaria parasites and experimental infection

The N67C parasite was initially obtained from the Malaria Research and Reference Reagent Resource Center (MR4, http://www.mr4.org/) and has been described previously^38^. Freshly thawed parasites were injected (*i.p*.) into naïve C57BL/6 mice to initiate infection. An inoculum containing 1 × 10^6^ infected red blood cells (iRBCs) suspended in 100 μl sterile phosphate buffer saline (PBS, pH 7.4) from the donor mice was injected intravenously (*i.v*.) into experimental mice. Parasitemia was assessed daily, from day 2 *p.i*., by examination of Giemsa-stained thin tail blood smears. The mice were monitored for symptoms and mortality (sacrificed after indicated days of infection or moribund as determined by an attending veterinarian). Body weight was measured and variation in body weight was presented as a percentage of the current body weight compared with the body weight before infection. Spleen tissues from anesthetized animals were removed, weighed, and then reserved for further analysis (Histopathology, ELISA, Western blot). Additional tissue samples (lung and liver) were removed for histopathology.

### Histopathology and immunofluorescence (IF)

Tissue samples (spleen, lung) were processed according to a standard protocol. Briefly, tissues were removed, immediately fixed in 10% neutral buffered formalin solution (Sigma, St. Louis, MO, USA), and blocked in paraffin for histological analysis. Tissue sections (5 µm) were stained with hematoxylin and eosin (H&E) and then evaluated by a pathologist in a blinded manner. Sections were examined under light microscopy using an Olympus BX51 microscope and photomicrographs were taken using an Olympus DP73 camera.

For IFA, tissue sections (5 µm) were heated to 60 °C for 1 h, deparaffinized with xylene washes, and rehydrated with alcohol-graduated washes. Heat-induced epitope retrieval (HIER) was performed by steaming the sections in DIVA Decloaker RTU solution (Biocare, Pacheco, CA, USA) for 20 min. After treatment with Protein Block solution for 30 min (Protein Block, Serum-Free RTU, Dako), primary antibodies were added and incubated for 1 h: Anti-F4/80 (Abcam, Clone A3–1) as a broad splenic Mɸ marker, anti-CD169/Siglec-1 (AbD Serotec, Clone MOMA-1) as a marginal zone metallophilic Mɸ marker, and anti-CD209b (Santa Cruz, Clone ER-TR9) as a marginal zone Mɸ marker. Biotinylated goat anti-rat IgG (Vector Laboratories, Burlingame, CA, USA) antibody was incubated for 1 h at RT after 3X washes, and signals were detected by incubating the sections for 30 min with streptavidin-conjugated AlexaFluor 594 (Life Technologies, Carlsbad, CA, USA). Erythrocyte autofluorescence was quenched with a solution of 10 mM CuSO_4_/50 mM ammonium acetate, pH 5. Sections were counterstained with DAPI (Vector Laboratories).

### ELISA and Western blot

Cytokines in supernatants of tissue homogenates were measured using commercial ELISA kits (R&D Systems, Minneapolis, Minnesota, USA). For Western blot, total proteins were extracted using the Zoom 2D Protein Solubilizer Kit (Invitrogen, San Diego, California, USA) supplemented with Protease Inhibitor Cocktail, Phosphatase Inhibitor Cocktail (Sigma) and Protease Inhibitor Tablets (Roche Applied Sciences, Indianapolis, Indiana, USA). Protein extracts (25 µg) were separated on a SDS-PAGE gel, transferred to a PVDF membrane (Roche Diagnostics, Indianapolis, IN, USA), and probed with indicated primary antibodies and corresponding secondary antibody prior to chemiluminescent detection (SuperSignal West Pico Chemiluminescent Substrate, Pierce, Rockford, Illinois, USA). Rabbit anti-cleaved-caspase-3 (#9664), anti-cleaved-caspase-8 (#895), anti-granzyme B (#4275), mouse anti-cleaved PARP mAb (#9648), anti-rabbit IgG (#7074), and anti-mouse IgG (#7076) were purchased from Cell Signaling Technology (Danvers, MA); Mouse anti β-actin mAb (AC-74) was from Sigma-Aldrich (St. Louis, MO).

### Assessment of vascular integrity

The integrity of the endothelial vasculature was investigated using Evans blue (EB) dye as previously reported^75^. Mice were injected *i.v*. with 0.2 mL of 1% EB solution (Sigma-Aldrich) on day 6 *p.i*. One hour later, mice were sacrificed and perfused intracardially with 10 mL of PBS. Tissue samples (spleen, lung, liver, kidney, brain, gut) were harvested and weighed, and EB extravasation was evaluated as an index of increased capillary permeability by measuring absorbance at 610 nm after formamide treatment (1 mL) for 24 h at 56 °C.

### Preparation and analysis of bronchoalveolar lavage (BAL)

BAL was performed by inserting a catheter into the mouse’s trachea after euthanasia^76^. The bronchoalveolar compartment was flushed with 1 mL of PBS, and then centrifuged at 300 g for 10 min. Cells in the pellet were counted using a hemocytometer (Cellometer, Nexcelom Bioscience, Lawrence, MA, USA), and protein content was measured using Pierce 660 nm Protein Assay (ThermoFisher Scientific, Waltham, MA, USA).

### Flow cytometry

Live cells were counted using a hemocytometer (Cellometer, Nexcelom Bioscience) and trypan blue exclusion. Isolated leucocytes in spleen from individual animals were stained with Fixable Viability Dye (eBioscience, San Diego, CA, USA) and an appropriate combination of mAbs specific for surface and intracellular staining in the presence of anti-mouse CD16/CD32 blocking antibodies. Intracellular staining was performed after cells were fixed and permeabilized using Perm-Fix solution (eBioscience). For intracellular expression of IFN-γ and TNF-α in T cell population, *ex vivo* CD3/CD28 activation was performed using Dynabeads® Mouse T-Activator in the presence of Brefeldin A according to manufacturer’s instructions (Life Technologies). All samples were acquired on a FACSCalibur (BD Immunocytometry Systems and analyzed using FlowJo software (TreeStar).

### Statistical analysis

Data were analyzed using GraphPad Prism 6 (GraphPad Software, La Jolla, California, USA). Comparisons between 2 groups were performed using Mann-Whitney test, and multiple groups were compared using Kruskal-Wallis test. For survival curves, a log-rank Mantel-Cox test was used. Values of *p* < 0.05 were considered statistically significant.

## Electronic supplementary material

Supplementary Figures
