# Supplementary material for: Mechanism of splenic cell death and host mortality in a Plasmodium yoelii malaria model
Source: Sci Rep. 2017 Sep 5;7:10438. doi: 10.1038/s41598-017-10776-2 (PMC5585408; doi:10.1038/s41598-017-10776-2)
Supplement: Supplementary file 1 — Supplementary Figures [file 41598_2017_10776_MOESM1_ESM.pdf]

Mechanism of splenic cell death and host mortality in a *Plasmodium yoelii* malaria model

Norinne Lacerda-Queiroz[1], Nicolas Riteau[2], Richard T. Eastman[1], Kevin W. Bock[3], Marlene S. Orandle[3], Ian N. Moore[3] Alan Sher[2], Carole A. Long[1], Dragana Jankovic[2], & Xin-zhuan Su[1]*

[1]Laboratory of Malaria and Vector Research, and [2]Laboratory of Parasitic Diseases, [3]Infectious Disease Pathogenesis Section of Comparative Medicine Branch, National Institute of Allergy and Infectious Diseases, National Institutes of Health, Bethesda, Maryland 20892-8132, USA

Supplementary Information:

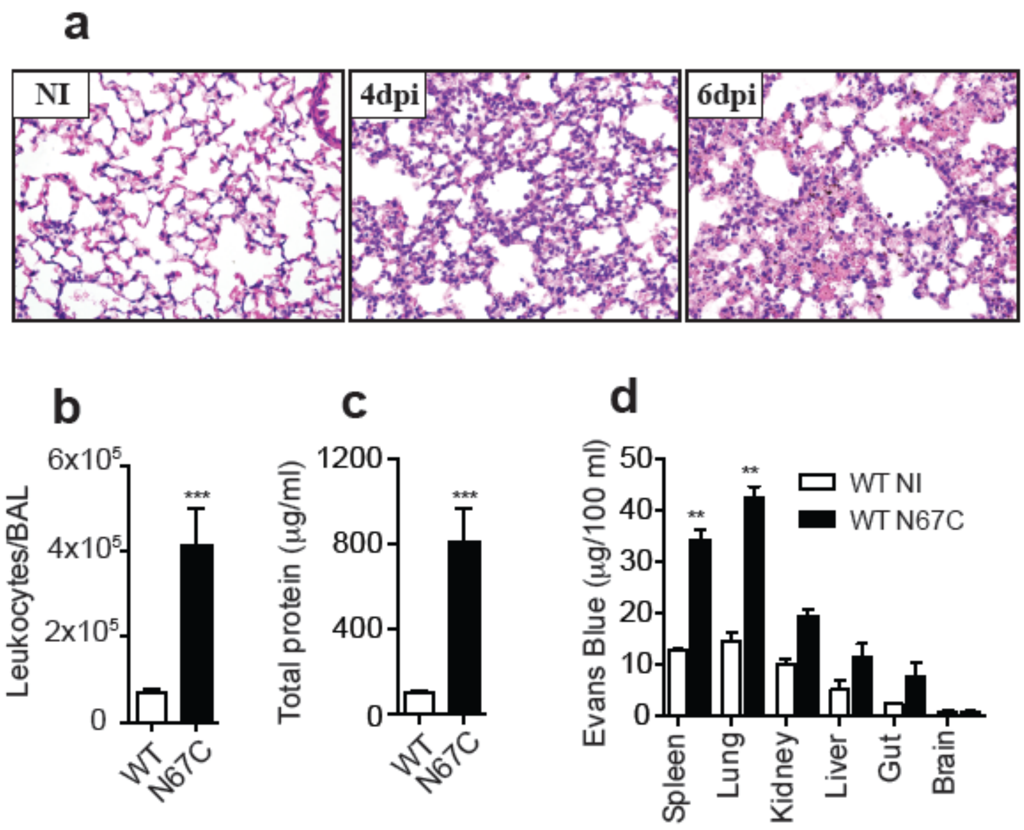

Supplementary Figure 1. N67C infection induces a systemic inflammatory process in the lung. **(a)** Representative images (400X magnification) of hematoxylin and eosin (H&E) stained lung tissue sections from non-infected (NI) and N67C-infected mice at days 4 and 6 *p.i.* (**b**,**c**) Number of leukocytes **(b)** and total protein **(c)** in the bronchoalveolar lavage (BAL) from NI and N67C-infected mice on day 6 *p.i.* **(d)** Quantification of Evans Blue in the tissue parenchyma of NI and N67C-infected mice on day 6 *p.i.* The results in **b**–**d** are expressed as mean ± SEM (3–5 mice) and are representative of two experiments. Significant tests: Mann–Whitney U test, $^{**}p<0.01$, $^{***}p<0.001$.

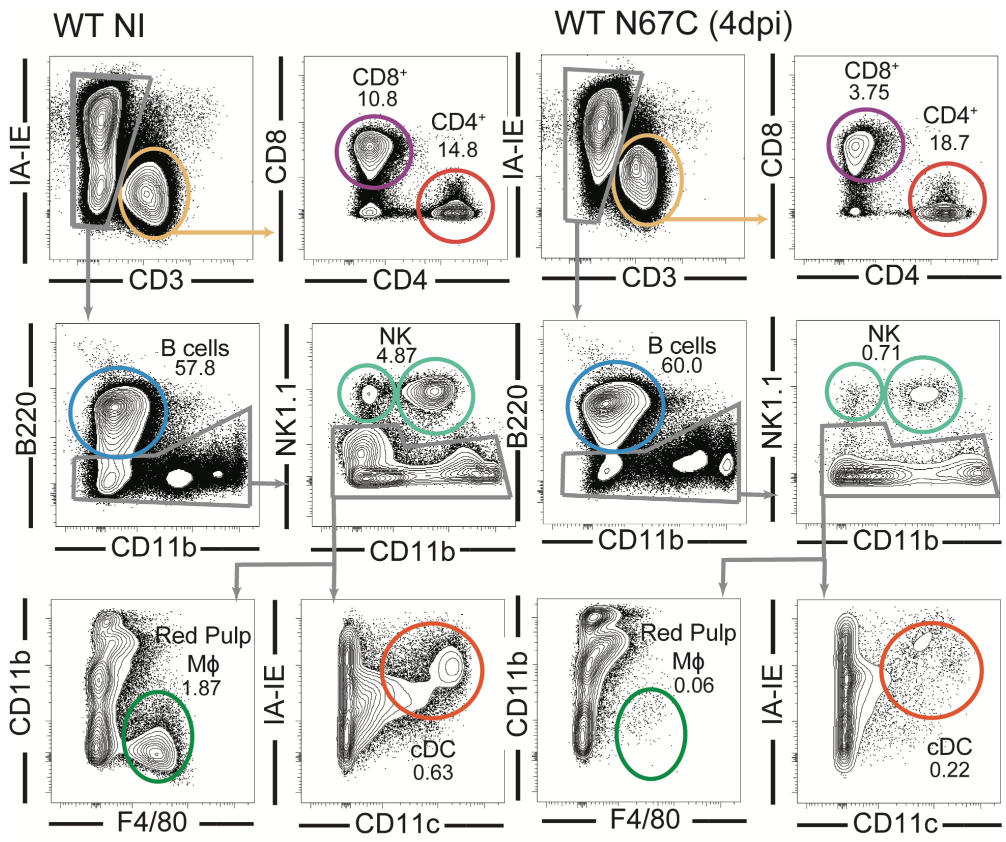

Supplementary Figure 2. Representative flow cytometry plots of non-infected (NI) and N67C-infected mice day 4 *p.i.* Markers for different splenic cell populations are as marked, and gating strategies used to identify cellular subsets in the spleen tissue after selection of live+ CD45+ cells are shown. The number for each gated population is the frequency of specific cell (%) in the whole population.

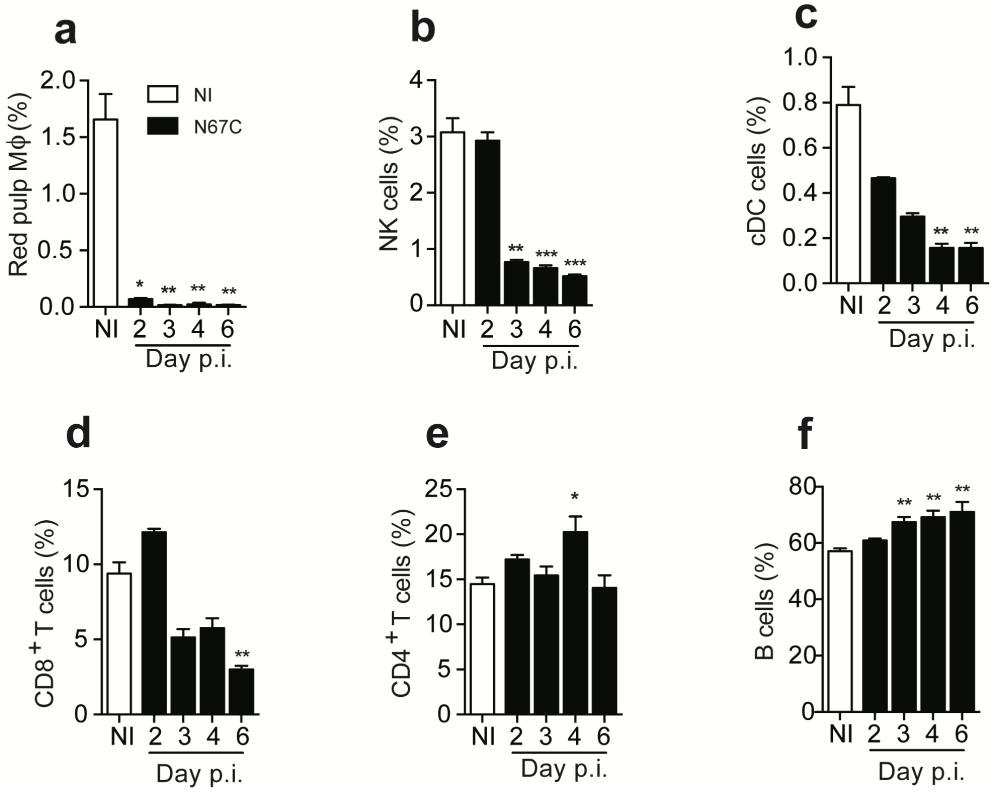

Supplementary Figure 3. Percentages of cell populations in the spleens of infected mice. Cell populations including red pulp macrophages (Mϕ) **(a)**, natural killer (NK) cells **(b)**, conventional dendritic cells (cDC) **(c)**, CD8+ T cells **(d)**, CD4+ T cells **(e)**, and B cells **(f)** were analyzed using flow cytometry after staining for specific cell markers. The percentage of cell count is expressed as mean ± SEM (from 3–5 mice); Kruskal-Wallis test, *p<0.05, ***p*<0.01, and ****p*<0.001 (compared to NI group).

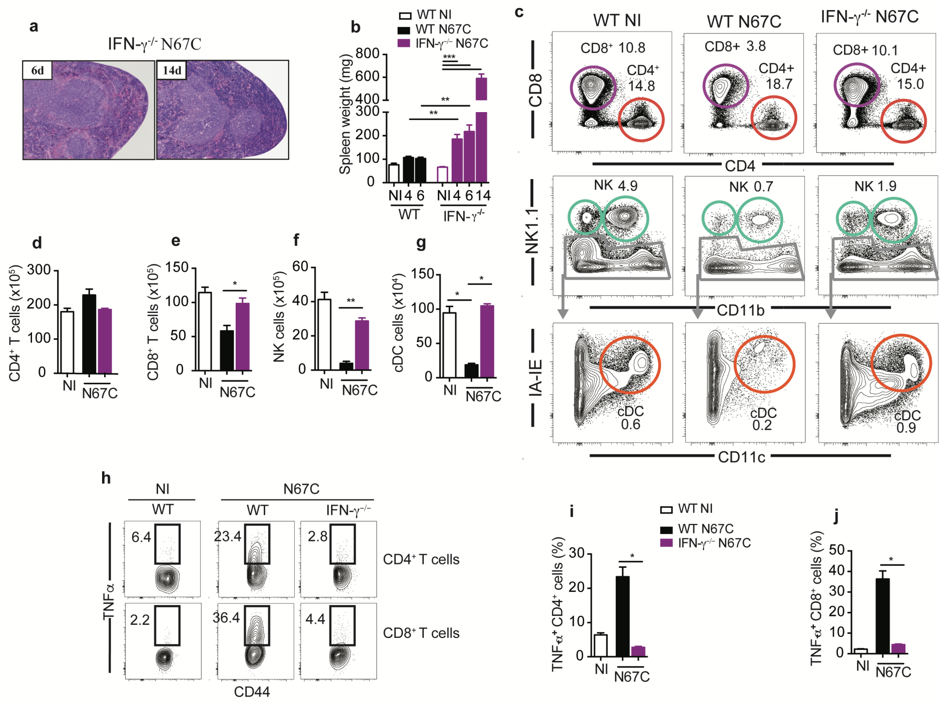

Supplementary Figure 4. Comparison of cell populations in wild type (WT) and IFN-$\gamma^{-/-}$ mice after N67C infection. **(a)** Hematoxylin and eosin (H&E) stain of spleen tissues of IFN-$\gamma^{-/-}$ mice day 6 and 14 *p.i.* **(b)** Spleen weights of uninfected (NI) and infected WT and IFN-$\gamma^{-/-}$ mice day 4, 6, and 14 *p.i.* **(c–g)** Flow cytometry analysis of cell populations in the spleens of NI WT and infected WT or IFN-$\gamma^{-/-}$ mice day 4 *p.i.*, including CD4+ and CD8+ T cells **(d,e)**, NK cells **(f)**, and cDC cells **(g)**. **(h–j)** Percentages of CD4+ and CD8+ T cells expressing intracellular TNF-α in NI WT and infected WT or IFN-$\gamma^{-/-}$ mice day 4 *p.i.* Graphs are expressed as mean ± SEM (from 3–5 mice) and are representative of two experiments; Kruskal-Wallis test, *p<0.05, ***p*<0.01, and ****p*<0.001 (compared to NI group).

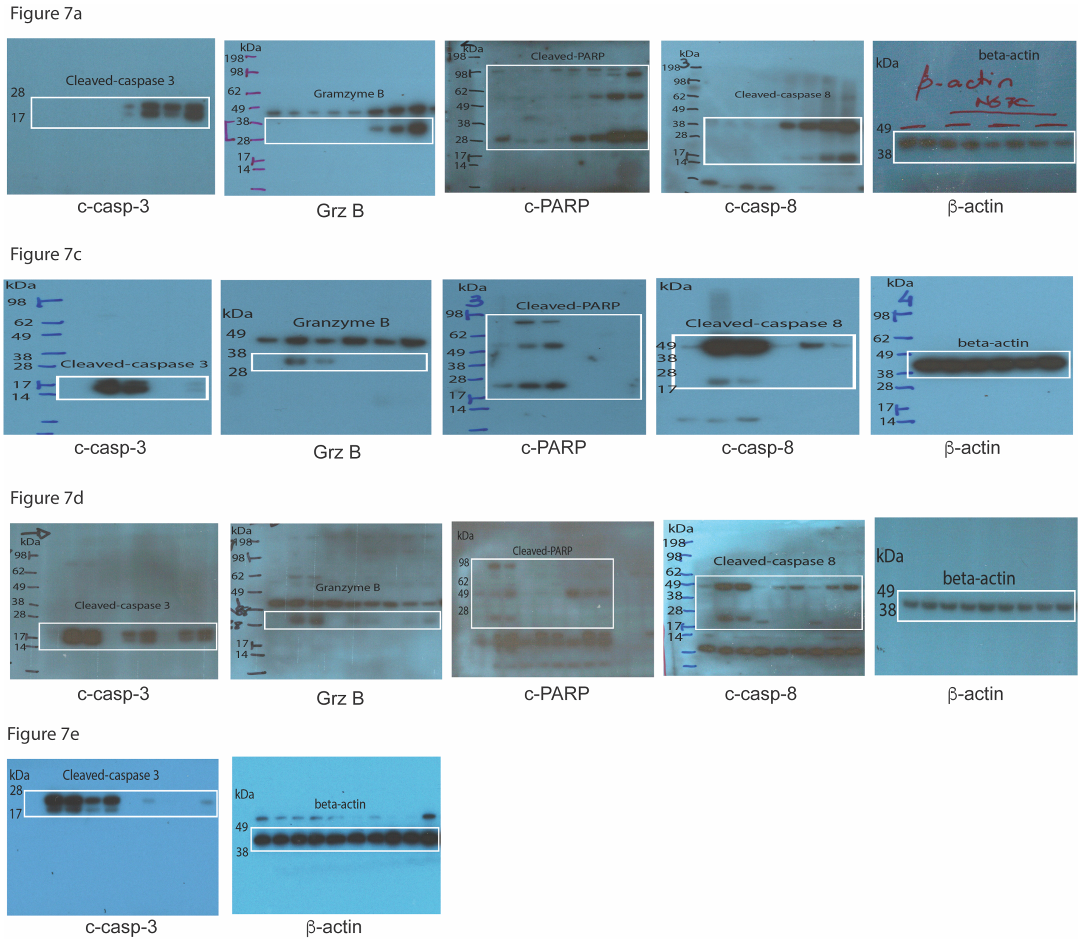

Supplementary Figure 5. Original Western blot images for panels in Figure 7. The areas within the white boxes are cropped and presented in Fig 7a, 7c, 7d, and 7e.

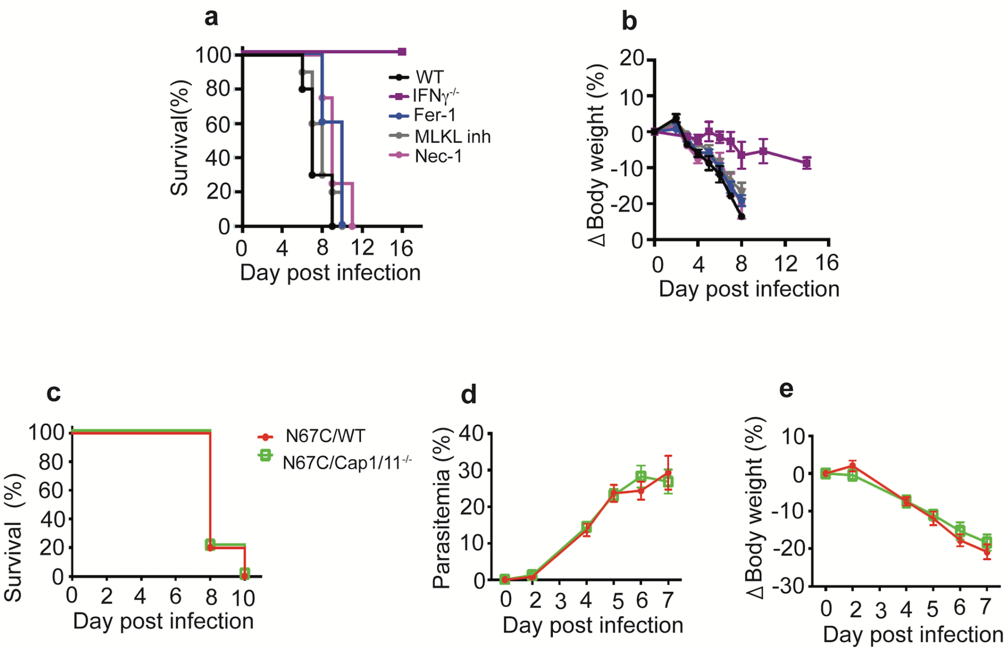

Supplementary Figure 6. Effects of inhibition of selected cell death pathways and double deficiencies of caspase 1 and caspase 11 on parasitemia or host survival. **(a)** Survival rates of WT, IFN-$\gamma^{-/-}$, or mice treated with necrostatin-1 (Nec-1), GW806742X (MLKL inhibitor), and ferrostatin-1 (fer-1) after N67C infection. **(b)** Body weight of the mice in **a**. **(c–e)** Survival rate **(i)**, parasitemia **(j)**, and body weight **(k)** of WT and caspase-1/caspase-11 double KO mice after N67C infection.

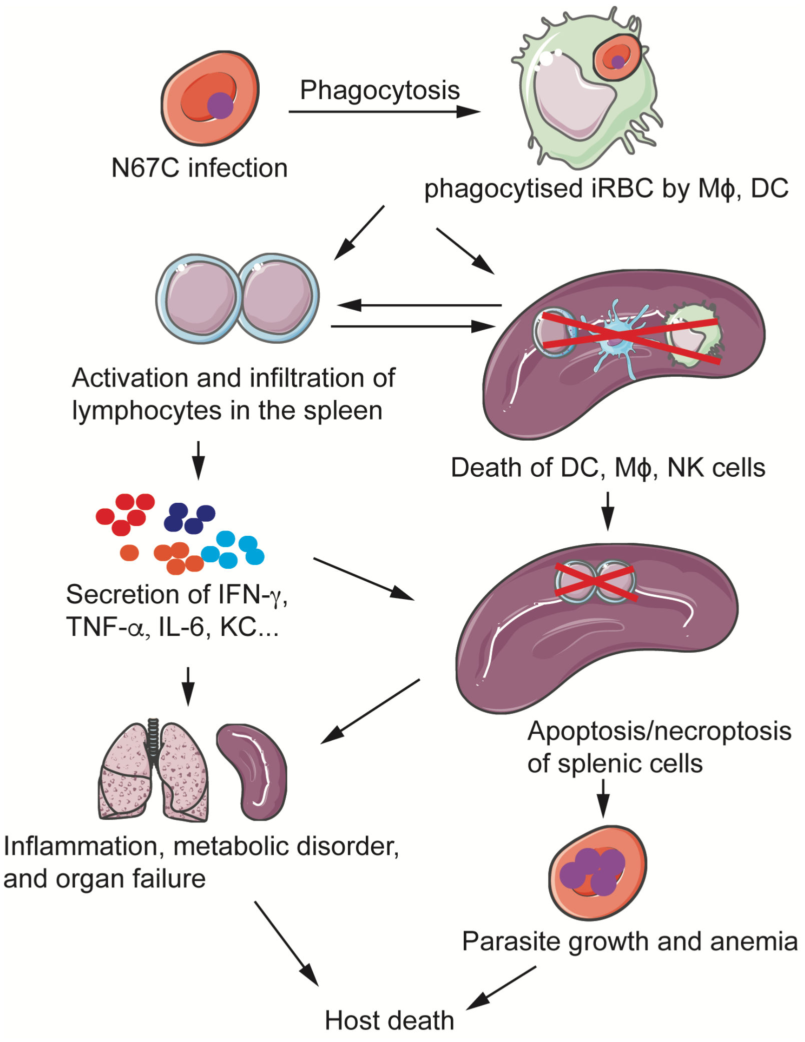

Supplementary Figure 7. Summary of sequential events leading to splenic cell apoptosis and host death. N67C infection results in early (day 2) leukocyte infiltration in the spleen, lung, and other organs, and the death of red pulp Mϕ, NK, and DC cells through unknown mechanisms. These events further activate lymphocytes, particularly T cells, to produce pro-inflammatory chemokines and cytokines, including IFN-γ, and TNF-α, leading to more extensive cellular death in the spleen, BM, and possibly other tissues. The mechanism of cell death was mediated mostly by apoptosis and possibly necroptosis; although we cannot totally rule out the involvement of other cell death pathways. The death of phagocytic cells and the failure of spleen function allow

continuous growth of parasites. The cause of host death is likely due to multiple factors, including excessive inflammatory responses, a compromised immune system, organ failure, and metabolic disturbances such as hypoglycemia and lactic acidosis that we did not study here. This figure was created by the senior author (X.Su) using Servier Medical Art images under free Creative Commons (license CC-BY) at: http://smart.servier.com/.
